# Heads or tails first? Evolution of fetal orientation in ichthyosaurs, with a scrutiny of the prevailing hypothesis

**DOI:** 10.1186/s12862-023-02110-4

**Published:** 2023-04-18

**Authors:** Feiko Miedema, Nicole Klein, Daniel G. Blackburn, P. Martin Sander, Erin E. Maxwell, Eva M. Griebeler, Torsten M. Scheyer

**Affiliations:** 1 Staatliches Museum für Naturkunde Stuttgart, Rosenstein 1, 70191 Stuttgart, Germany; 2 Institut für Biologie, Fachgebiet Paläontologie, Hohenheim University, Wollgrasweg 23, 70599 Stuttgart, Germany; 3 Paläontologisches Institut, Universität Zürich, Karl Schmid-Strasse 4, Zurich, CH-8006 Switzerland; 4 Abteilung Paläontologie, Institut für Geowissenschaften, Universität Bonn, Nußallee 8, 53115 Bonn, Germany; 5 Dept. of Biology and Electron Microscopy Facility, Trinity College, Hartford, CT 06106 USA; 6 Dinosaur Institute, Natural History Museum of Los Angeles County, 900 Exposition Boulevard, Los Angeles, CA 90007 USA; 7 Institut für Organismische und Molekulare Evolution, Universität Mainz, Hanns-Dieter- Hüsch-Weg 15, 55128 Mainz, Germany

**Keywords:** Ichthyopterygia, Fetal orientation, Viviparity, Asphyxiation risk, Aquatic birth, *Mixosaurus*, *Stenopterygius*

## Abstract

According to a longstanding paradigm, aquatic amniotes, including the Mesozoic marine reptile group Ichthyopterygia, give birth tail-first because head-first birth leads to increased asphyxiation risk of the fetus in the aquatic environment. Here, we draw upon published and original evidence to test two hypotheses: (1) Ichthyosaurs inherited viviparity from a terrestrial ancestor. (2) Asphyxiation risk is the main reason aquatic amniotes give birth tail-first. From the fossil evidence, we conclude that head-first birth is more prevalent in Ichthyopterygia than previously recognized and that a preference for tail-first birth likely arose in derived forms. This weakens the support for the terrestrial ancestry of viviparity in Ichthyopterygia. Our survey of extant viviparous amniotes indicates that fetal orientation at birth reflects a broad diversity of factors unrelated to aquatic vs. terrestrial habitat, further undermining the asphyxiation hypothesis. We propose that birth preference is based on parturitional mechanics or carrying efficiency rather than habitat.

**Supplementary Information:**

The online version contains supplementary material available at 10.1186/s12862-023-02110-4.

## Introduction

Ichthyopterygia is a clade of Mesozoic reptiles adapted to marine life. One of the numerous adaptations that helped the group radiate in, or even invade the marine realm is viviparity [1]. There is direct evidence for viviparity in at least ten different ichthyosaur genera spread across the group’s phylogeny, as gravid females are known from (roughly in phylogenetic hierarchy): *Chaohusaurus*, *Mixosaurus*, *Cymbospondylus*, *Besanosaurus*, *Shonisaurus*, *Qianichthyosaurus*, *Ichthyosaurus*, *Leptonectes*, *Stenopterygius*, *Maiaspondylus* and *Platypterygius* [2–14]. It is therefore commonly accepted that all members of Ichthyopterygia were viviparous [1]. *Stenopterygius* and *Ichthyosaurus* are so far the only genera with multiple described gravid specimens, and the sample of *Stenopterygius* is vastly larger than that of any other taxon [14]. This situation has led to the fact that most of the knowledge on reproduction and prenatal development in ichthyosaurs is based on this genus [14–16].

Viviparity in ichthyosaurs was first proposed in 1842 [17], following the discovery of the first gravid female of *Stenopterygius* in 1749 (SMNS 2). The earliest discussions usually pertained to whether the preserved smaller specimens were fetuses or possible gastric contents resulting from predation or cannibalism [18]. However, since the late 1980s the consensus is that the material is fetal for almost all of the specimens studied [14, 19]. Böttcher [14] published a comprehensive study of 35 gravid specimens of *Stenopterygius* (most of which are currently considered *S. quadriscissus* [20]). He studied litter size, fetal size, *in utero* position, and fetal orientation at birth. Litter size in *Stenopterygius* is highly variable and does not seem to be related to maternal size, in contrast generally to extant reptiles [14, 21]. However, this study was published in German [14], and the language choice, as well as publication in a relatively poorly distributed journal, may have caused incomplete understanding of the matter and a subsequent over-generalization from this study in the literature. One of the major observations in *Stenopterygius* was their preferred birth position, which was predominantly tail-first (fetal skull facing anteriorly with respect to the mother) [14]. This led to the generalization that in ichthyosaurs birth orientation was preferentially tail-first [12]. However, the basal ichthyosauromorph *Chaohusaurus* was subsequently described with two fetuses in situ, one of which presumably in the birth canal, showing clear head-first orientation (fetal skull facing caudally with respect to the mother) [2].

### Birth orientation across Ichthyopterygia and its proposed terrestrial origin

Motani and colleagues proposed that viviparous reproduction likely evolved in the terrestrial ancestors of Ichthyopterygia and that *Chaohusaurus*, as one of the basal-most ichthyosaurs, still retained the ancestral head-first birth position [2]. Their paper incorrectly inferred from Brinkmann’s [8] account that (based on the knowledge available at the time) *Mixosaurus* already showed a tail-first parturition preference, and left out the nuances concerning the tail/head birth positions of *Stenopterygius*. This led to the hypothesis that birth orientation switched early in the evolutionary history of ichthyosaurs [2]. Subsequent observations in *Cymbospondylus* (LACM DI 158109: [9]) show that, at least in some cases, head-first birth did occur in some Triassic ichthyosaurs more derived than *Chaohusaurus*. In other ichthyosaurs, orientation is distributed as follows (counts refer to maternal specimens): *Besanosaurus*: 1 specimen, the holotype, *in utero*, orientation suggesting tail-first birth [10, 22]; *Shonisaurus*: 1 specimen, unknown orientation [3, 7]; *Qianichthyosaurus*: 1 specimen *in utero* orientation suggesting tail-first birth [11]; PMS, NK pers. observ.; *Ichthyosaurus*: 3 specimens, all inconclusive [12, 23] and FM pers. observ.; *Leptonectes*: 1 specimen, inconclusive [13]; *Maiaspondylus*: 1 specimen, inconclusive [4]; and *Platypterygius*: 1 specimen *in utero* orientation suggesting tail-first birth [24].

The assumption that derived ichthyosaurs should show a preference for tail-first birth was based on the idea of the prevalent “increased asphyxiation risk” hypothesis, which infers that head-first orientation of the fetus reduces the risk of drowning/asphyxiation during the birth process [19, 25]. The hypothesis possibly arose after undocumented and likely unreasonable observations of long (multiple weeks long?) births in beluga whales *Delphinapterus leucas* [26, 27]. Asphyxiation risk has subsequently been cited to explain why the fetus of the Eocene protocetid whale *Maiacetus* showed an *in utero* orientation suggestive of head-first birth [28]. The authors hypothesized that the head-first orientation in *Maiacetus* showed the ancestral artiodactyl condition and proposed that *Maiacetus* possibly gave birth on land and that subsequent obligatorily marine cetaceans would have given birth tail-first [28].

Here we add to this discussion by documenting the orientation of fetuses in three specimens of *Mixosaurus* cf. *cornalianus*, one of which was previously studied [8]. *Mixosaurus* is a fully marine adapted Middle Triassic ichthyosaur already displaying dorsal and caudal fins, a streamlined body and flippers [29]. It is ubiquitously found to be an early diverging member of the Ichthyosauria [30, 31]. The new observations on fetus orientation in *Mixosaurus* prompted us to review the literature on ichthyosaur reproduction and add nuances and taphonomic concerns regarding the study of parturition in gravid female ichthyosaurs. We provide a short review of the reproductive strategies of other marine reptiles and briefly compare them to that of ichthyosaurs. Likewise, we survey birthing positions in certain extant viviparous mammals and reptiles to test the postulate that tail-first births are necessarily an adaptation to giving birth in water. Lastly, we propose a novel hypothesis on when and why parturition orientation may have switched from head-first to tail-first in Ichthyopterygia.

## Results

### PIMUZ T 4830

**Fig. 1:**
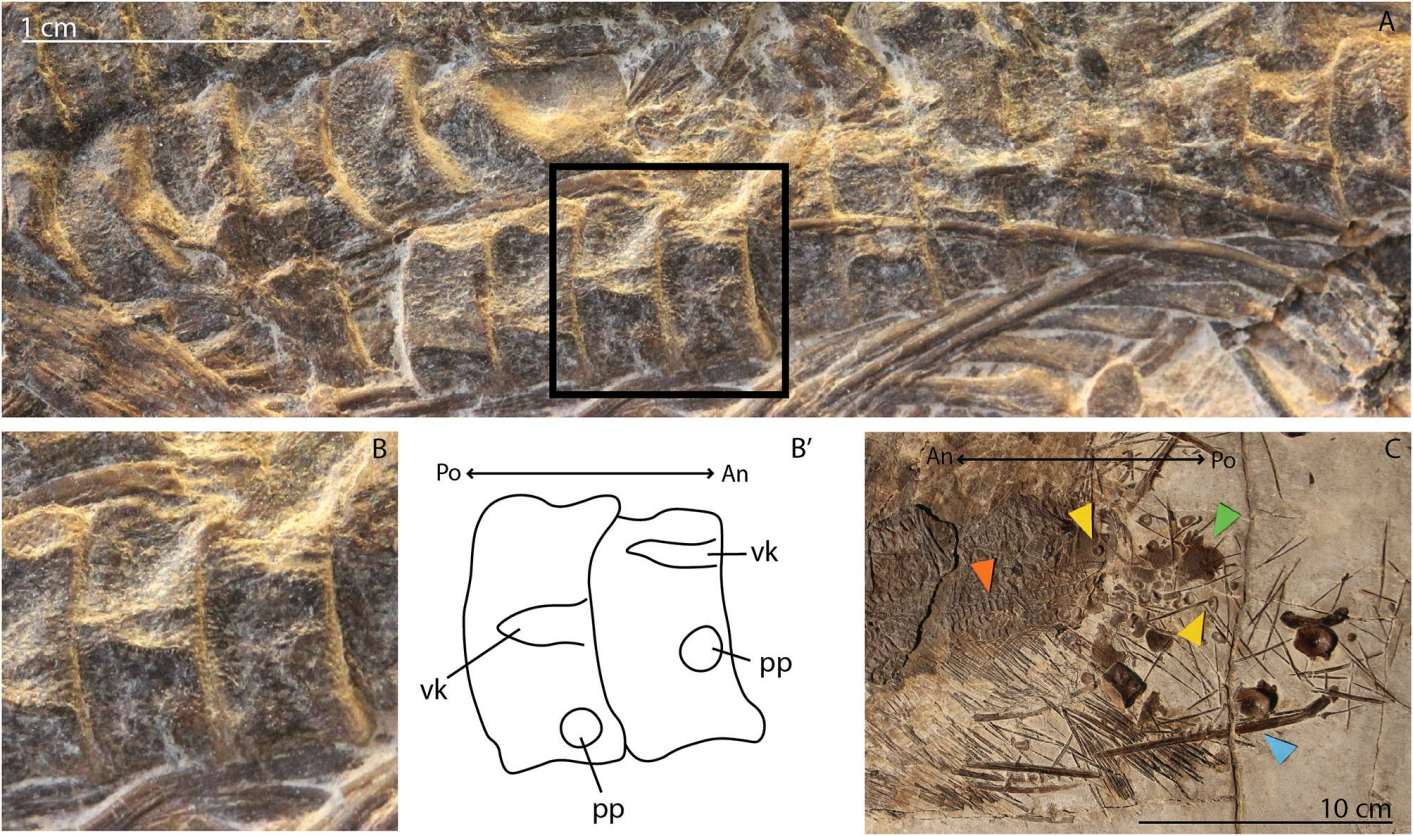
Details and overview of the in situ fetal material associated with female *Mixosaurus cornalianus* PIMUZ T 4830: A, detail of the articulated fetal vertebral columns and associated ribs in the posterior reproductive tract; B, magnified view of the two vertebrae shown in the box in A (An and Po denote fetal anterior and posterior); C, overview of the posterior portion of the maternal trunk, including the two fetal articulated vertebral columns and associated anterior girdle, limb and lower jaw elements (An and Po denote maternal anterior and posterior). Interpretations denoted by an apostrophe after their respective letter. Abbreviations: pp, parapophysis; vk, ventral keel

PIMUZ T 4830 is a complete gravid specimen of *Mixosaurus* containing two fetal vertebral columns in the maternal body cavity and disarticulated fetal cranial and postcranial material outside the maternal cavity. In the more ventrally situated fetal vertebral column, two centra show clear rib facets. This vertebral column is preserved in ventral view, which means these facets should be parapophyses. The rib facets lie on the part of the lateral centrum surface closest to the maternal tail. The positioning therefore shows that the fetus is oriented facing the maternal tail, suggesting head-first parturition (Fig. 1A, B). This inference is further corroborated by the orientation of the associated fetal ribs. The ribs are usually directed posteriorly in laterally flattened ichthyosaur specimens e.g., [32], which would also suggest head-first positioning in this fetus. Furthermore, fetal cranial, pectoral girdle and forelimb material is only observed just posterior to the end of the birth canal (Fig. 1A, C) outside of the mother’s body. All fetal pelvic and hind limb elements are found above the mother on the slab. There is therefore strong evidence that these fetuses were positioned head-first towards the cloaca.

### PIMUZ T 2262

**Fig. 2:**
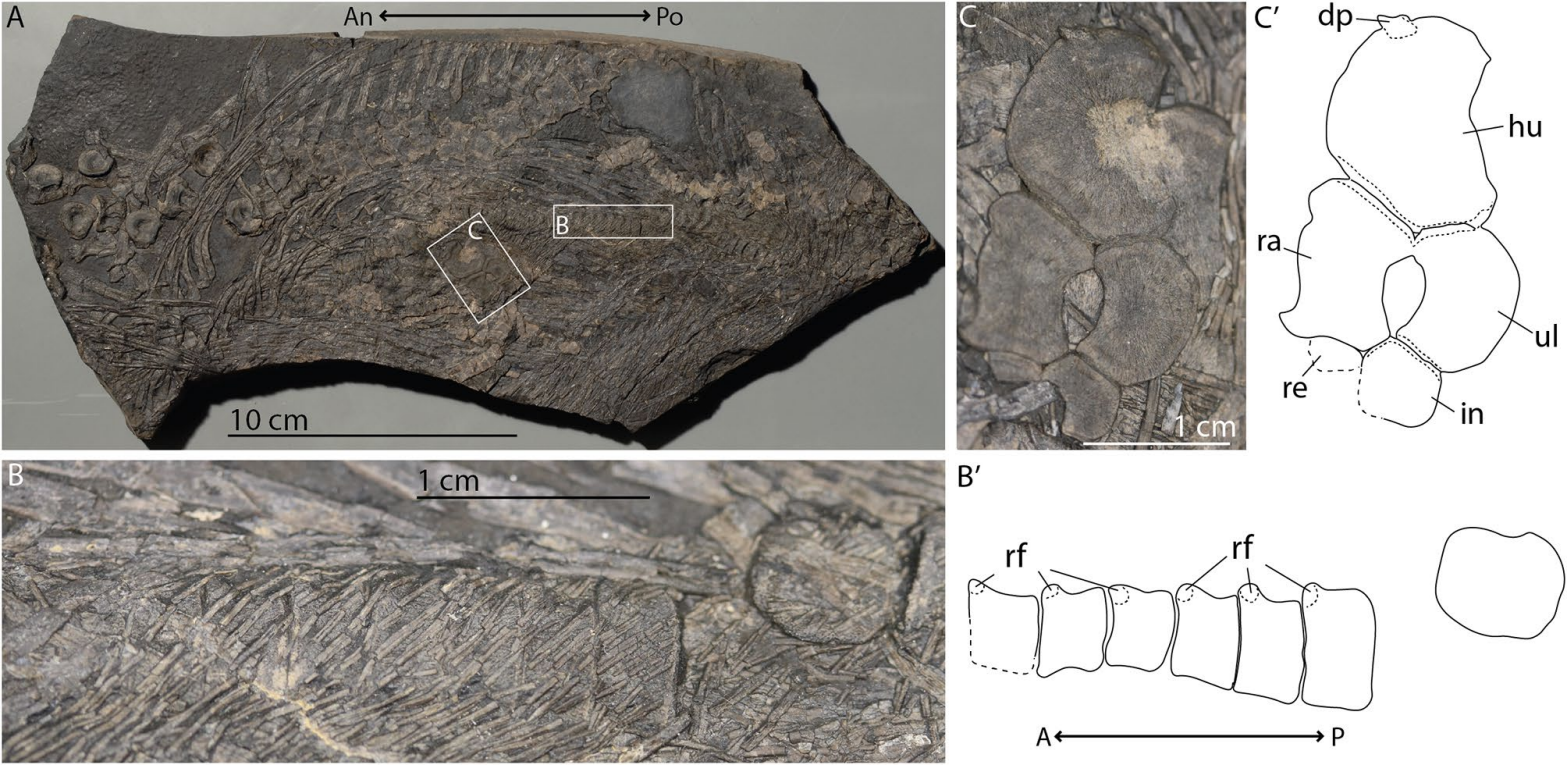
Overview and details of the trunk of female *Mixosaurus* sp. PIMUZ T 2262 and associated in situ fetal material: A, complete overview of the specimen (An and Po denote maternal anterior and posterior); B, detail of the articulated fetal vertebral column also visible in A; C, detail of the fetal proximal anterior limb also seen in A. Interpretations denoted by an apostrophe after their respective letter. Abbreviations: dp, dorsal process; hu, humerus; in, intermedium; ra, radius; re, radiale; rf, rib facet; ul, ulna

PIMUZ T 2262 is a maternal anterodorsal trunk fragment of *Mixosaurus* containing two fetal (semi)articulated vertebral columns and a proximal limb fragment including a humerus, radius and ulna. The more dorsally situated of the two vertebral columns shows clear rib facets. It is unclear in which view the column is visible, but likely in ventral view, because of the overlapping associated gastralia (Fig. 2A-B). The rib facets are directed towards maternal anterior *in utero*, suggesting a tail-first birth orientation. The fetal flipper is not clearly associated with either vertebral column, but its angle relative to its associated vertebral column and the maternal vertebral column also indicate tail-first *in utero* orientation (Fig. 2C).

### PIMUZ T 1902

**Fig. 3:**
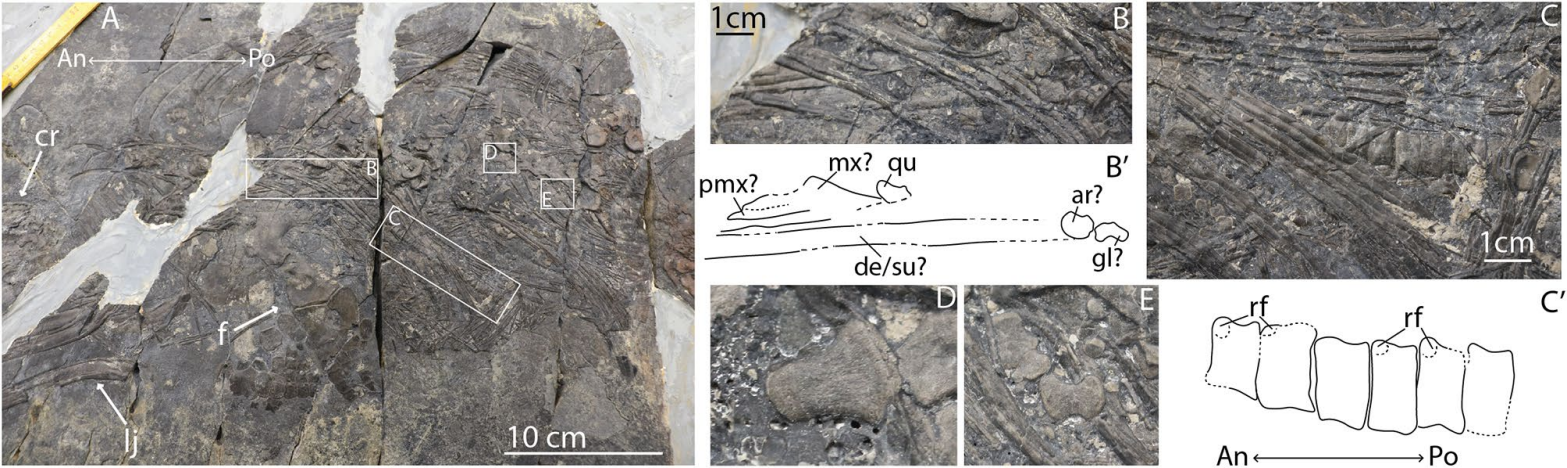
Overview of the trunk of female *Mixosaurus cornalianus* PIMUZ T 1902 and in situ associated and articulated fetal material: A, complete overview of the specimen (An and Po denote maternal anterior and posterior); B, detail of the maternal trunk containing fetal cranial elements (An and Po denote fetal anterior and posterior); C, detail of the maternal trunk containing the articulated fetal vertebral column; D, fetal tibia; E; fetal fibula and astragalus. Interpretations denoted by an apostrophe after their respective letter. Abbreviations: ar, articular; cr, maternal cranium; de/su, dentary surangular; f, maternal left forelimb; gl, glenoid; lj, maternal lower jaw; mx, maxilla; pmx, premaxilla; qu, quadrate; rf, rib facet

PIMUZ T 1902 is a complete gravid specimen of *Mixosaurus* containing a single fetus, which includes fragmentary cranial material, an articulated vertebral column, two fragmentary anterior forelimbs (including at least a radius and proximal autopodial elements) and a disarticulated posterior limb. Fetal cranial material is located just dorsally of the maternal scapula in the maternal ribcage. The fetus therefore likely lies *in utero* (Fig. 3). More posteroventrally lies an articulated vertebral column with associated forelimbs. It is unclear what surface of the vertebral centra is visible, but rib facets are present on some of them. Rib facets are positioned towards maternal anterior (Fig. 3). More posteriorly, there is a disarticulated femur and tibia. These three observations strongly suggest an orientation *in utero* for subsequent tail-first birth.

### General notes

Litter size is small in all three studied specimens of *Mixosaurus*, being one in PIMUZ T 1902 and two in PIMUZ T 2262 and PIMUZ T 4830. The latter specimen possibly contains a third fetus; it is difficult to determine given the disarticulated nature of the preserved elements. Cranial fetal material is present in PIMUZ T 1902 and PIMUZ T 4830. The high degree of ossification of the dermal skull bones (especially the skull roof elements) as well as an advanced degree of ossification in the chondrocranium suggests a developmental stage comparable to stage 4 of *Stenopterygius* [15], meaning the fetuses are at a stage where prenatal development is largely finished and birth is imminent. Thus, in conclusion, the fetuses in PIMUZ T 1902 and PIMUZ T 2262 display orientations *in utero* suggesting tail-first birth and those in PIMUZ T 4830 are in head-first presentation close to the cloaca.

### Reanalysis of birth orientation in *Stenopterygius*, based on data in Böttcher 1990

Litter size in *Stenopterygius* ranged from 1 to 11; the largest litter was observed in one female (MfN, number not provided), but many were observed to have only one fetus; the average was found to be 3.3 [14].

**Fig. 4:**
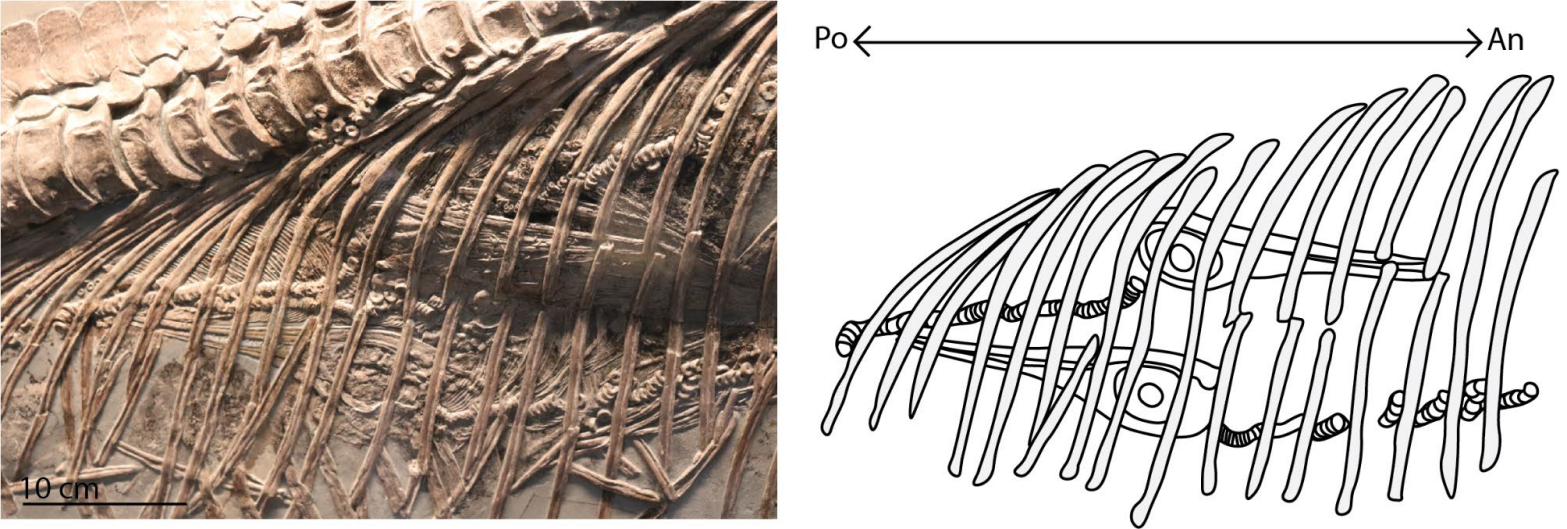
Detail of the trunk of gravid *Stenopterygius quadriscissus* (MHH 1981/33) and interpretative drawing showing stage 4 fetuses with both head-first and tail-first *in utero* orientations. This suggests that both birth orientations were present in a single gravid female (An and Po denote maternal anterior and posterior)

Based on the data Böttcher provides plus our own observations (suppl data 1) on *Stenopterygius* (SMNS 81961 and SMNS 80234), we calculate that in roughly 54% (22/41) of the preserved gravid females tail-first fetal birth canal orientation is observed, whereas in 15% (6/41) of cases head-first birth canal orientation is present (all associated fetuses clearly show the same orientation). The other cases (13/41) were deemed inconclusive, as the fetuses were not preserved in situ in the reproductive tract or were too disarticulated. Remarkably, in two cases (SMNS 16811 and MHH 1981/33), fetuses were preserved together in the maternal body cavity in both orientations (Fig. 4). Fetal orientations were not correlated with bed number in the Posidonienschiefer Fm.; both fetal orientations were recovered before and after the onset of oceanic warming associated with the early Toarcian Anoxic Event, but head-first birth seemingly increased in frequency following the onset of the event, with both cases of mixed litter orientation occurring after the onset. However, the difference in frequencies of both fetal orientation before and after this event was not significant (Fisher’s exact test, N_prior_ = 9, N_after_ = 8, p = 0.294). We are therefore cautious to conclude a climatic effect based on the limited data.

## Discussion

### Cranial orientation in *Mixosaurus*

Head-first birth position in the fetuses of PIMUZ T 4830 has been hypothesized previously based on the distribution of jaw elements towards the maternal pelvic girdle, in combination with the recovery of caudal vertebrae dorsal to the adult pectoral girdle [8]. A subsequent discussion of this [8] study mistakenly reported that the fetuses had “cranially oriented” skulls, i.e., in a position for being born tail-first [2]. The two new specimens of gravid *Mixosaurus* here described do indeed display *in utero* tail-first orientation. Given the small sample size (n = 3 females), it is difficult to conclude anything on preferred birth orientation in *Mixosaurus*, but it is at least not strictly tail-first. It is in our opinion unlikely that the fetuses of PIMUZ T 1902 and PIMUZ T 2262 would have been able to turn *in utero* as they are in an advanced stage of development when compared to *Stenopterygius*.

### Evolution of orientation at birth across Ichthyopterygia

Our reanalysis of Böttcher’s data supports the tail-first birth orientation preference reported in the original study; in fact tail-first birth predominated by a factor of 3.6:1 in *Stenopterygius*.

Whether or not our observations reflect preferred orientation in the sample of *Stenopterygius* at birth depends on the following questions: does either birth position lead to greater pregnancy complications, and if so, is our sample biased towards the disadvantageous orientation? To neither question do we have a fully satisfactory answer at this time. However, in modern odontocetes, both birth orientations are recorded and even though they have a large preference for tail-first birth, neither position has been observed to result in major complications during birth [33–36]. This leads us to the hypothesis that even though we may not be seeing the true frequency distribution of parturition and *in utero* orientations, we are unlikely to see a strong bias towards complicated pregnancies in the fossil record of *Stenopterygius*.

The other factor that we have to take into account is potential fetal turning *in utero*. Fetal turning is unfortunately not well studied in any viviparous animal. Extant lepidosaurs would be the closest living viviparous relatives of ichthyosaurs. In viviparous squamates (as in their oviparous counterparts), fetuses are tightly curled through lateral flexion, forming a compact rounded mass tightly enclosed within the fetal membranes [37, 38]. In contrast, ichthyosaur fetuses are only curled up early in development (i.e., stage 1 *Stenopterygius* [15]); subsequently they are stretched out (elongated) in a cranial/caudal direction [2, 4, 9, 14]. Given the differences in uterine morphology, fetal turning in ichthyosaurs can therefore not be ruled out. That said, the fact that there is a set moment in prenatal development in which the fetus goes from a curled to a straight morphology could mean that this is the period in which the embryo needs to acquire its orientation for subsequent parturition, possibly due to limited uterine space. It has also been suggested that the fetus stiffens at this moment [4]. To presume that *in utero* orientation therefore equals birth orientation is plausible, and we will use this assumption throughout the rest of the discussion.

**Fig. 5:**
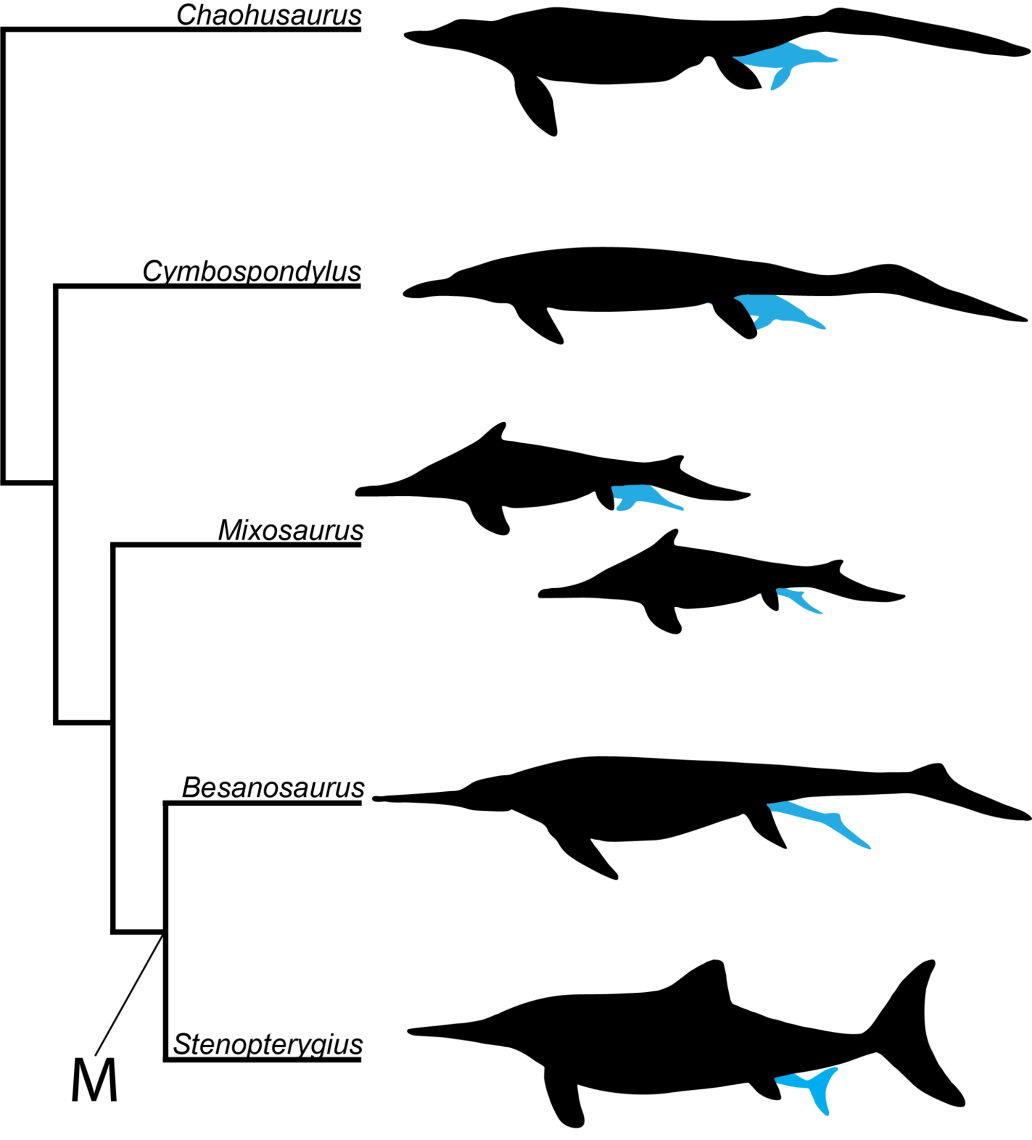
Simplified phylogenetic bracketing of five ichthyopterygians on the basis of recent phylogenetic analyses and their preferred birth orientation. Early-diverging forms such as *Chaohusaurus* and *Cymbospondylus* both have one gravid specimen with head-first birth, *Mixosaurus* has a 66%/33% based on three specimens, and Merriamosauria (phylogenetic node denoted with M) likely have a slight preference for tail-first birth based on the sample of *Stenopterygius* and the fact that all other Merriamosauria show tail-first birth or are inconclusive (note: the relative phylogenetic position of *Cymbospondylus* and *Mixosaurus* is debated; compare e.g., [30, 31] (silhouettes adapted from: *Chaohusaurus* [2]; *Cymbospondylus* [87]; *Mixosaurus* [29]; *Besanosaurus* [84]; *Stenopterygius* [89]

In all ichthyosaurian taxa studied apart from *Stenopterygius*, the sample size is far too small to assess preferred birth orientation. However, in general head-first birth is more common in the small sample size of more basal Triassic taxa (*Chaohusaurus*, *Cymbospondylus*), whereas all specimens of the derived pelagic Merriamosauria (e.g., *Besanosaurus*, *Qianichthyosaurus*, *Ichthyosaurus*, *Stenopterygius, Platypterygius*) in which orientation can be assessed show tail-first presentation in their single specimen or average sample, as does *Mixosaurus*. We therefore hypothesize that a (slight) preference of tail-first parturition is more prevalent from the base of the Merriamosauria and that likely all more basal members either had a slight head-first birth preference or no preference either way (Fig. 5). Tail-first presentation preference originated therefore much later and is likely much less pronounced than previously hypothesized. This also makes the hypothesis that head-first orientation is an evolutionary remnant of terrestrial ancestors less likely, but not impossible. The current evidence neither supports nor rejects that possibility. Moreover, nothing is currently known about the reproductive strategies of the early diverging ichthyosauriforms Omphalosauridae (= Nasorostra [39]), and the sister taxon of ichthyosaurs, Hupehsuchia. Inferences about the ancestral reproductive condition of ichthyosaurs can therefore not yet be made. All in all, we should not be surprised to find either orientation in subsequent new discoveries across the phylogeny, given what we now know about preference variability.

### The ichthyosaur reproductive strategy compared to other extant and extinct taxa

Viviparous reproduction is a widespread pattern among reptiles, both extant and extinct. Phylogenetic analyses have concluded that viviparity has evolved independently in more than 115 separate clades of lizards and snakes and that about 20% of the extant species are viviparous [40–43]. Among pre-Cenozoic Reptilia, recent analysis inferred a minimum of six independent origins of viviparity [44, 45], represented by mesosaurs, sauropterygians, ichthyopterygians, choristoderes, mosasauroids, and the Cretaceous lizard *Yabeinosaurus.* Evidence of one additional origin has since been adduced in Triassic marine archosauromorphs [46, 47]. Finally, the marine thalattosuchian crocodylomorphs of the Jurassic and Cretaceous also have been posited to have given birth to live young [48]. Each of these reptilian groups is considered below, in comparison to ichthyosaurs.

### Mesosauridae

Possibly the oldest known viviparous amniotes were the mesosaurs. Several adults of the genus *Mesosaurus* have been found in very close association with neonatal/early juvenile specimens [44, 49]. There is also one specimen with a clear *in utero* fetus [49]. However, there is also a fetus or neonate found in a curled position with no parental association. This fetus also had a structure which was tentatively interpreted as an egg-tooth [49]. No eggshells of mesosaurs have so far been identified. The fetus without parental association is curled, similar to oviparous and viviparous squamates or egg-laying reptiles, whereas the fetus *in utero* was more loosely coiled [49].

### Eosauropterygia

Within Eosauropterygia, viviparity is documented in the fossil record by *in utero* fetuses in the Triassic pachypleurosaur *Keichousaurus hui* [50, 51] and in the Late Cretaceous polycotylid plesiosaur *Polycotylus latipinnus* [52]. In addition, an isolated fetus without an eggshell of the Middle Triassic pachypleurosaur *Neusticosaurus* [53, 54] and isolated fetuses of the pachypleurosaur *Keichousaurus*, also not sheltered by an eggshell, are documented in the fossil record [51]. Isolated fetuses lacking eggshells are also known in the Middle Triassic nothosaur *Lariosaurus* [55]. So far, evidence for viviparity in Placodontia is absent.

Fetuses of *Keichousaurus* are curled up *in utero*, whereby the neck region generally wraps around the trunk. They are generally spaced sequentially in the posterior maternal trunk, similar to modern lepidosaurs [50, 51]. Recently, published evidence shows that the fetuses emerge head- (or rather neck) first, as the curled posterior neck and pectoral girdle appear first out of the birth canal in one specimen [51]. This is another example of an aquatic animal giving birth head-first in the water column.

The *Polycotylus* fetus is too disarticulated to infer a degree of curling or birth position [52].

### Extant lepidosaurs

As stated, the major difference between ichthyosaur fetuses and those of extant lepidosaurs lies in their conformation during development, whereby lepidosaurs are generally curled *in utero* and ichthyosaurs are not [37, 38]. The ichthyosaur uncurled morphology has been attributed to either maternal uterine restrictions or more likely the stiffening of the vertebral column over ossification [4]. Either way, the situation suggests that the relationship of fetuses to their fetal membranes differs from that of viviparous lepidosaurs; in the latter, the fetal membranes and oviduct are closely wrapped around the fetus [42, 56], precluding major reorientation. One end of ichthyosaurian fetuses is often found close to the maternal pectoral girdle [9, 14, 22], suggesting that the reproductive tract lay along the entire dorsal side of the trunk, as is typical of reptiles [57]. In viviparous squamates, the neonate often emerges curled from the maternal cloaca still encased in its fetal membranes; thus, there is no fetal orientation *per se*. In viviparous sea snakes [58, 59], the neonates commonly emerge from the cloaca head-first, no longer surrounded by their fetal membranes. In the aquatic lizard *Shinisaurus crocodilurus*, video records reveal that each neonate exits the cloaca explosively with no consistent orientation, and rapidly swims away (Appendix 1).

### Mosasauroidea

The aigialosaur *Carsosaurus* unsurprisingly shows an *in utero* condition closely matching extant viviparous squamates [60]. In the specimen studied, fetuses lie sequentially curled up with regular spacing, which is typical of squamates [57, 60]. The authors suggested a tail-first birth preference in their interpretation. However, it is more likely that fetuses emerge in a curled position, as in other viviparous squamates.

### Choristodera

The exact phylogenetic position of Choristodera is uncertain, but it is regularly recovered as sister taxon to either Lepidosauromorpha or Archosauromorpha [61]. Within Choristodera, we only have evidence for a viviparous reproductive strategy in the Early Cretaceous taxon *Hyphalosaurus baitaigouensis*. Two gravid females have been found: one containing fetuses in the body cavity and one containing, and associated with, embryos in eggs [62, 63]. Furthermore, unassociated soft-shelled eggs with embryos have been found [64] as well as isolated but curled up apparent fetuses (pers. observation by PMS at PMOL). It is possible that the species had both oviparous and viviparous populations, but it may be more likely that it is actually two closely related species displaying both reproductive strategies as occurs regularly in modern squamates [40, 43]. The embryos/fetuses lie curled up both *in ovo* and *in utero*, although *in ovo* there seems to be more curving [63, 64]. The fetuses are distributed across the maternal body cavity sequentially anteroposteriorly and possibly pair-wise [63]. The fetus closest to the cloaca is oriented such as suggesting head-first birth [63]. The authors hypothesized this may have complicated the birth, calling tail-first birth the norm in aquatic amniotes. However, this inference is questionable, given their sample size of gravid *Hyphalosaurus* (1 published gravid specimen), and the data presented here.

### Archosauromorpha

Only one viviparous archosauromorph taxon has so far been discovered: the Middle Triassic *Dinocephalosaurus* [46, 47]. The main evidence is a single gravid specimen containing a single semi-articulated fetus [47]. The fetus is slightly coiled, similar to squamates but not as extensively, and in a head-first *in utero* position, suggestive of a possible tail-first birth. Moreover, there is an isolated expelled fetus attributed as cf. *Dinocephalosaurus* [46]. The specimen’s taphonomy and lack of a calcified eggshell is indirect further evidence for viviparity in the taxon. The specimen is heavily curled similar to squamate fetuses [46]. Given the difference in articulation between the two fetal specimens, it is difficult to state with certainty if head-birth or tail-first birth is the norm in *Dinocephalosaurus*.

### Mammalia

Outside of Cetacea, aquatic and semi-aquatic mammals do not show a clear preference in fetal orientations at birth. In Sirenia, both head-first and tail-first births have been recorded [65–67]. Such is also the case for hippopotami [27, 68, 69] (Appendix 1) (which can give birth in water or on land), as well as semi-aquatic mammals that give birth on land, such as sea otters [70] and pinnipeds [71–73]. Further, tail-first births clearly are not simply an adaptation to the aquatic habitat, given their presence in such large terrestrial mammals as elephants [69, 74, 75] (Appendix 1), as well as domestic pigs [76] and smaller mammals such as members of Chiroptera [77] and Sciuridae (Appendix 1).

As for cetaceans, while tail-first deliveries predominate, they are far from universal. For example, one literature summary noted that the rate of head-first births was 7% in the killer whale *Orcinus orca* and 14% in captive belugas, with head-first deliveries being labeled a “natural variation” [78] (also see Appendix 1). In Odontoceti as a whole, birth orientation is indeed predominantly tail-first, although head-first births are recorded and were generally observed not to be more complicated or stressful than tail-first births [33–36, 79]. In Mysticeti, the preference is relatively unknown, due to difficulties in observation. However, data based on whaling expeditions in the first half of the twentieth century indicate that *in utero* orientation is at least close to 50/50 in the genus *Balaenoptera* (fin whales and blue whales) and observations of birth in the genus *Megaptera* (humpback whale) are generally tail first [33]. Little is known about fetal turning in whales, so it is hard to say if the whaling data reflect preferred birth orientation.

Overall, birth orientation in mammals may reflect a variety of factors other than habitat, including shape and relative size of the late-term fetus, speed of parturition, length of the umbilical cord, and orientation of the uterus and vagina [27, 33, 80]. In a detailed review of mammalian parturition, tail-first births in cetaceans [71] are attributed to the fact that the head and thorax are both bulky and rigid (given the absence of a flexible neck), and the posterior body and tail mobile and light. Under the influence of gravity the head and neck sink to the lower, cranial end of the uterus, while the tail is oriented posterodorsally towards the cervix. As a result, during parturition, the tail exits the uterus and cloaca first [71].

### Ichthyosaur parturition and the “increased asphyxiation risk” hypothesis

The asphyxiation hypothesis is, given the evidence, not well-supported: comparative data on extant taxa across amniotes show no clear relationship between birth orientation and habitat of the kind one would predict from this hypothesis (see Appendix 1). If fetuses born head-first were indeed prone to increased asphyxiation risk in secondarily aquatic tetrapods, we would expect to see a higher preference for tail-first births early in the evolutionary history of all aquatic, viviparous clades, due to strong stabilizing selection for this trait. This is not really the case (Appendix 1); we therefore deem the “increased asphyxiation risk” hypothesis to be unsupported by the data.

Placental mammals that are born head-first generally share the characteristics that they have a flexible neck, long appendicular elements and a wide pelvic girdle. The peristaltic movement of the uterus and vagina push on the pelvic girdle of the fetus. This is mechanically convenient as the pelvic girdle is one of the widest parts of the fetal body. Moreover, early expulsion of the head and neck ensures that these will not be in an awkward position, or even at risk of damage, if the head gets stuck in the birth canal. Ichthyosaurs and cetaceans morphologically share the evolution of a streamlined body, including the loss of a discrete neck and fusion, or shortening of the cervical vertebral column. This makes the cervical region in these clades less vulnerable during parturition. Moreover, the pelvic area in cetaceans and ichthyosaurs is extremely reduced over evolutionary history, making the fetal cranium larger than the pelvis. It is therefore mechanically and energetically advantageous to peristaltically push on the cranium. We therefore hypothesize that peristaltic expulsion mechanics during parturition favor a tail-first birth in cetaceans and ichthyosaurs, which would explain the parturition preference switch over evolutionary history in the two clades. This hypothesis would also explain the preferred birth orientation in the eosauropterygian *Keichousaurus* [51]. The neck and cranium are clearly the more vulnerable parts in the fetus, and the pelvic and pectoral areas are of similar size. For *Keichousaurus* it would therefore be advantageous to give birth head- (or rather neck) first.

Alternatively, it is possible that with the loss of the discrete neck and reduction of the pelvic girdle and hind limb, head-first or tail-first orientation are somewhat equal in terms of maternal energy use and danger to the fetus. In this case, maternal stress induced from the orientation during pregnancy is the main selection pressure for fetal orientation. For example, having the cranium directed anteriorly *in utero* (tail-first parturition) could have been slightly more advantageous than cranial posterior *in utero* orientation because in thunniform ichthyosaurs (and maybe cetaceans), the maternal center of mass would not be displaced as far from the anteriorly positioned center of buoyancy (the lungs) if the heavily ossified fetal crania were oriented anteriorly rather than posteriorly, thus reducing maternal energy expenditure on trim control [81].

## Conclusions

Three specimens of pregnant *Mixosaurus* were (re)examined. They show tail-first parturition preference in two cases (PIMUZ T 1902 and PIMUZ T 2262) and head-first parturition preference in the third case (PIMUZ T 4830). So far, the proposed hypothesis was that ichthyosaurs gave birth tail-first because of asphyxiation risk of the neonate during birth in the aquatic medium. Moreover, the head-first birth recorded in the early diverging ichthyosaur *Chaohusaurus* was proposed to be a remnant of the terrestrial origin of viviparity for the ichthyosaur clade. With the new data on *Mixosaurus*, the recently discovered head-first *in utero* orientation of *Cymbospondylus* and a review of the known literature on ichthyosaur *in utero* and parturition orientation, we propose that a slight preference for tail-first parturition possibly arose at the base of the Merriamosauria and that more basal ichthyopterygians either had a head-first preference or little preference. We want to stress that we should not be surprised to find either parturition orientation in future finds across phylogeny.

Moreover, the “increased asphyxiation risk” hypothesis is an unlikely explanation for birth preference based on our review of parturition preferences of modern amniotes. We propose two new hypotheses on why ichthyosaurs might have switched birth preference, namely: (1) it may have been mechanically advantageous to push on the cranium rather than the pelvis during ichthyosaur birth, due to gradual decrease of pelvic size, reduction of a discrete neck and increased body streamlining in parvipelvian ichthyosaurs; (2) *in utero* head-first orientation during pregnancy, which leads to tail-first birth, could have been advantageous energetically or in terms of trim control.

We advise caution when assessing parturition preference in fossil viviparous taxa as it is uncertain what percentage of the sample shows birth complications, e.g., still births *in utero*, and the potentially large percentage of complicated births (including the non-preferred parturition orientation).

## Materials and methods

### Specimens of gravid *Mixosaurus* examined

The three specimens of *Mixosaurus* were collected from the Besano Formation at the Monte San Giorgio locality during scientific excavations in the 20th century led by Emil Kuhn-Schnyder and Bernhard Peyer respectively. The locality is at the Swiss-Italian border and has recently become a UNESCO World Heritage site. The Besano Formation is Anisian-Ladinian (Middle Triassic) in age and has yielded many well-preserved vertebrate fossils [82–84]. See Furrer [85]and Röhl et al. [86] for an overview of the stratigraphy and paleoenvironmental interpretation of the formation.

The formation has yielded over 100 skeletons of the abundant genus *Mixosaurus*; however, gravid females are relatively rare. In addition to the material previously described (PIMUZ T 4830: [8]), we identified two further specimens of *Mixosaurus* in the PIMUZ collection: PIMUZ T 2262, and PIMUZ T 1902. Both were labeled as gravid females, but seemed to have shifted away from collective memory. PIMUZ T 2262 was rediscovered by the former curator Heinz Furrer and subsequently prepared; PIMUZ T 1902 was rediscovered by the first author and prepared for this study.

The orientation of the fetuses was inferred using the methodology outlined in Klein et al. [9]. Ichthyosaur vertebral centra have rib facets on the anterior side of the lateral surfaces [87, 88], and in Triassic ichthyosaurs, these become differentiated early enough in development to be useful in orienting fetuses relative to the mother. Therefore, if an articulated vertebral column is preserved in lateral view, the relative position of the rib facets indicates the anteroposterior orientation of the fetus. We use the following terminology to describe fetal orientation: tail-first/caudal orientation means that the cranium of the fetus is directed towards the maternal anterior and the tail towards maternal posterior (and vice versa for head-first/cranial orientation). This emphasizes the orientation at birth to avoid confusion throughout the manuscript.

### Assessment of ichthyosaur birth orientation and comparison

In order to discuss reproductive strategy and birth orientation in Ichthyopterygia as a whole, we re-use the data compiled by Böttcher [14] on *Stenopterygius.* We re-examined the *Stenopterygius* specimens in the SMNS, GPIT, MHH and NHMUK collections mentioned in the paper and added the two specimens SMNS 81961 and SMNS 80234 to our sample. We also review all other published gravid ichthyosaur specimens. We compare the ichthyosaur birthing strategy to other extinct and extant viviparous reptiles based on the literature. Lastly, to assess the validity of the asphyxiation risk hypothesis we compile published data on birth orientation preference in extant viviparous amniotes living in aquatic and terrestrial habitats.

### Terminological note

Up to this point, *in utero* specimens of ichthyosaurs have mostly been referred to as embryos in the paleontological literature (apart from Klein et al., 2020 who also use “fetus” instead of “embryo”). We suggest to adopt the term fetus in future. This is more in congruence with the terminology of other viviparous vertebrates in which a conceptus is referred to as embryo up to the onset of organogenesis, and referred to as a fetus from that point until birth. We have therefore used “fetus” throughout the manuscript, since all referred specimens have started ossification and therefore all organs are inferred to have begun development.

We have also adopted the term Ichthyopterygia sensu [30]. This ensures we have a standard term for the taxonomic unit including all ichthyosaurs and *Chaohusaurus*, but excluding Hupehsuchia and Omphalosauridae (Nasorostra) [39] of which we do not know anything regarding reproductive strategy.

## Electronic supplementary material

Below is the link to the electronic supplementary material.

Supplementary Material 1

Supplementary Material 2

## Data Availability

All specimens are available for study in their respective repositories. Contact the curators of marine reptiles at the SMNS (Dr. Erin Maxwell, co-author of this study), PIMUZ (Dr. Christian Klug) and Hauff Museum Holzmaden (Rolf Hauff) for specimen study.

## Institutional abbreviations

AGM: Anhui Geological Museum, Hefei, China
GPIT: Palaeontological Collection of Tübingen University, Germany
LACM: Natural History Museum of Los Angeles County, Los Angeles, USA
MfN: Museum für Naturkunde Berlin, Germany
MHH: Urweltmuseum Hauff Holzmaden, Germany
NHMUK: Natural History Museum United Kingdom, London, UK
PIMUZ: Paläontologisches Institut und Museum der Universität Zürich, Switzerland
PMOL: Paleontological Museum of Liaoning, Shenyang, China
SMNS: Staatliches Museum für Naturkunde Stuttgart, Germany
