# Supplementary material for: Heads or tails first? Evolution of fetal orientation in ichthyosaurs, with a scrutiny of the prevailing hypothesis
Source: BMC Ecol Evol. 2023 Apr 18;23:12. doi: 10.1186/s12862-023-02110-4 (PMC10114408; doi:10.1186/s12862-023-02110-4)
Supplement: Supplementary file 2 — Supplementary Material 2 [file 12862_2023_2110_MOESM2_ESM.docx]

Appendix 1. The following internet URLs link to videos of parturition in selected, viviparous mammals and reptiles. The videos document various mammals (chiefly aquatic and semi- aquatic forms). They indicate that birth positions do not correlate well with habitat. Likewise, as shown, in viviparous squamate reptiles, the offspring commonly emerges from the female while still encased in its fetal membranes (see the chameleon and the viper videos below). When the membranes break during parturition (as in the two semi-aquatic species represented below), either the head, the tail, or a body coil can emerge first from the maternal cloaca, followed immediately by the rest of the neonate. Thus “birth position” in squamates is not consistent or significant.

MAMMALS

Hippopotamus *(Hippopotamus amphibius)*

Head-first:

<https://www.youtube.com/watch?v=DQ5qBjjaWN8&ab_channel=WhereWildThingsRoam>

<https://www.youtube.com/watch?v=ll8BznaO9WM&ab_channel=DallasZoo>

Tail-first:

<https://www.youtube.com/watch?v=IG51RLu6yEs&ab_channel=Worldly>

<https://www.youtube.com/watch?v=nTl_H9CzNGk&ab_channel=NTVAnimals>

Elephant

Tail-first *(Elephas maximus)*:

[https://www.youtube.com/watch?v=97CRwd_U2FU&ab_channel=MasonElephantPark%26Lodge](https://www.youtube.com/watch?v=97CRwd_U2FU&ab_channel=MasonElephantPark%252526Lodge)

<https://www.youtube.com/watch?v=wg2cI-Pj2bM&ab_channel=bridgevideo>

<https://www.youtube.com/watch?v=wxPYG2VYGmU&ab_channel=KOCO5News>

Tail-first *(Loxodonta africana):*

<https://www.youtube.com/watch?v=MSxw6D6Wl4U&ab_channel=DavidXing>

Manatee (*Trichechus* *manatus*):

Head-first:

<https://www.youtube.com/watch?v=rDI_5Zv-5q0>

Rhinoceros *(Ceratotherium simum)*

Head-first:

<https://www.youtube.com/watch?v=IcVB5AcLm8Q&ab_channel=ANIMALSGIVINGBIRTH>

Beluga whale *(Delphinapterus leucas)*

Tail-first:

<https://www.youtube.com/watch?v=LXBzGf54-Ik&ab_channel=VancouverAquarium>

<https://www.youtube.com/watch?v=3rEolrMGkAs&ab_channel=GeorgiaAquarium>

Head-first:

<https://www.youtube.com/watch?v=-kA_DYtuqIc&ab_channel=NowThisNews>

<https://www.youtube.com/watch?v=Xyxi6roAnp0&ab_channel=SheddAquarium>

Humpback whale *(Megaptera novaeangliae)*

Tail-first:

<https://baleinesendirect.org/en/calving-humpback-caught-on-film-for-first-time/>

<https://www.youtube.com/watch?v=qVjRjEazdJs>

Orca (“killer whale”) *(Orcinus orca)*

Tail-first birth:

<https://www.youtube.com/watch?v=OW9kNWLF8Po&ab_channel=OnDemandNews>

Squirrel (*Sciurus* sp.)

Tail-first birth:

<https://www.youtube.com/watch?v=czBVPPOcq5A&ab_channel=Beautyofnature>

REPTILES

Chameleon *(Trioceros jacksonii*?*)*

<https://www.youtube.com/watch?v=adRNk6jjJnY&t=91s>

Gaboon viper (*Bitis* *gabonica*)

<https://www.youtube.com/watch?v=dx8uBPtFVoY&ab_channel=AndrewMaddox>

Chinese crocodile lizard ***(****Shinisaurus* *crocodilurus****)*** (birth under water)

<https://www.youtube.com/watch?v=eE3AvYqkNH0&ab_channel=JoeyMarkx>

<https://www.youtube.com/watch?v=YZvPPW9qX2Y&ab_channel=JoeyMarkx>

Green anaconda *(Eunectes* [notaeus](https://en.wikipedia.org/wiki/Eunectes_notaeus)*)* (birth under water)

<https://www.youtube.com/watch?v=oJfdTGevOrM>
